# Extraction, Purification, Characterization, and Antiangiogenic Activity of Acidic Polysaccharide from *Buddleja officinalis*

**DOI:** 10.1155/2020/5175138

**Published:** 2020-10-17

**Authors:** Xiaoteng Yan, Zhuan Yan, Qingping Xiong, Gaoqin Liu, Juanjuan Zhu, Peirong Lu

**Affiliations:** ^1^Department of Ophthalmology, The First Affiliated Hospital of Soochow University, Suzhou 215006, Jiangsu, China; ^2^Department of Ophthalmology, Affiliated Huai'an Hospital of Xuzhou Medical University, Huai'an 223002, Jiangsu, China; ^3^Department of Emergency, Huai'an First People's Hospital, Nanjing Medical University, Huai'an 223300, Jiangsu, China; ^4^Jiangsu Key Laboratory of Regional Resource Exploitation and Medicinal Research, Huaiyin Institute of Technology, Huai'an 223003, Jiangsu, China

## Abstract

Firstly, optimal parameters of crude polysaccharide from *Buddleja officinalis* were obtained as follows: ratio of water to raw material of 26 : 1, ultrasonic power of 240 W, ultrasonic time of 45 min, and ultrasonic temperature of 62°C. Secondly, acidic polysaccharide (APBOM) from *Buddleja officinalis* was successfully acquired with the yield of 9.57% by using DEAE-52 cellulose and Sephadex G-100 gel column chromatography. Then, we found that total polysaccharide content of APBOM was 94.37% with a sulfuric acid group of 1.68%, uronic acid content of 17.41%, and average molecular weight of 165.4 kDa. Finally, APBOM was confirmed to have significant antiangiogenic effects.

## 1. Introduction

Neovascularization is the common pathological basis and important clinical features of many eye diseases [1, 2], such as retinopathy of prematurity, diabetic retinopathy, age-related macular degeneration, and retinal vein occlusion. Currently, angiogenesis has been recognized as one of the high-risk factors of blinding eye disease [3]. Inhibition of ocular angiogenesis has become an important strategy with great therapeutic value for vision threatening ocular disorders [3, 4]. Nonspecific thermal laser photocoagulation of new blood vessels and intravitreal antivascular endothelial growth factor injection were a commonly used treatment for ocular angiogenesis [3]. However, these methods not only fail to completely abolish angiogenesis but may also damage the overlying outer retina and retinal pigment epithelium, thereby limiting their clinical application [5]. Therefore, it is essential to develop novel therapeutic drugs with high potency and low toxicity.

Acid polysaccharides refer to the polysaccharide containing acid groups collectively, such as sulfuric radical and uronic acid [6]. It was found that acid polysaccharides often have specific physicochemical properties due to the introduction of acid groups [7]. Compared with nonacidic polysaccharides, acidic polysaccharides tend to be more electronegative and less flexible, and their solubility also changes significantly [8]. These special physicochemical properties give acidic polysaccharides a number of unique pharmacological activities [9]. Inhibition of angiogenesis is one of the most important of them [9]. Currently, many acidic polysaccharides have been used to inhibit angiogenesis [9–12], which revealed significant efficacy and very few side effects.

*Buddleja officinalis* is the dried flower buds and inflorescences of *Buddleja officinalis* Maxim. [13]. As a traditional herbal medicine, *Buddleja officinalis* has been used to treat eye diseases in China for more than 1,000 years [14]. Pharmacological studies have shown that *Buddleja officinalis* has a significant effect on multiple ocular angiogenesis [15, 16], including cornea, conjunctiva, and retina. So far, however, its effective substances remain unclear. Excitingly, recent phytochemical studies have identified that *Buddleja officinalis* contains mainly polysaccharides, phenylethanoid glycosides, flavonoids, iridoids, and saponins [17]. In view of the significant efficacy of acidic polysaccharides in inhibiting angiogenesis, we hypothesized that the effective substances of *Buddleja officinalis* on treating ocular angiogenesis might be the acid polysaccharides contained in its body. Nevertheless, there is still a lack of reliable data to support the theory, which needs to be further confirmed. In order to test this hypothesis, here, we would systematically study the extraction, purification, characterization, and antiangiogenic activity of acidic polysaccharide (APBOM) from *Buddleja officinalis*.

## 2. Materials and Methods

### 2.1. Materials and Reagents

*Buddleja officinalis* was purchased from the Anhui Bozhou Herb Market (Bozhou, China), which was identified by Prof. Qingping Xiong from Huaiyin Institute of Technology, Huai'an, Jiangsu, China.

Dextrans with different molecular weight (*M*_*w*_) were from American Polymer Standards Co. (Colorado, USA). Monosaccharide (rhamnose, arabinose, fucose, xylose, mannose, glucose, and galactose) and uronic acid (glucuronic acid and galacturonic acid) standards were obtained from Shanghai Yuanye Bio-Technology Co., Ltd. (Shanghai, China). The 1-phenyl-3-methyl-5-pyrazolone (PMP) was purchased from Hangzhou Dayangchem Co. Ltd. (Hangzhou, China). DEAE-52 cellulose and SephadexG-100 were purchased from Whatman (Maidstone, Britain) and Amersham (Uppsala, Sweden), respectively. All other reagents used in this study were of analytical grade.

### 2.2. Preparation Process of Crude Polysaccharides (PBOM) from *Buddleja officinalis* by Ultrasonic-Assisted Extraction

PBOM was prepared by ultrasonic-assisted extraction. Briefly, the dried *Buddleja officinalis* was crushed to a 60-mesh sieve. The powder was fully degreased and decolorized using the anhydrous ethanol reflux method. After degreasing and decolorizing, the pretreatment powder was completely vacuum-dried at 50°C. The dried pretreatment powder was mixed with proper deionized water. Under set ultrasonic power, temperature, and time conditions, an ultrasonic extractor (BILON-T650CTG, Guangzhou Bilang Instrument Co., Ltd.) was used to extract PBOM. After extraction, the mixture was centrifuged at 5000 rpm for 20 min. The herb residues were reprocessed twice according to the above technological parameters. All extracted supernatants were collected and combined. These extracts are concentrated to a suitable volume and fully deproteinized by the Sevag method [18]. The sample solution after deproteinization was stirred vigorously and mixed with three times volume of absolute ethanol. Then, the mixture was placed overnight at 4°C to precipitate the polysaccharides. The precipitate was separated by centrifugation at 5000 rpm for 20 min and lyophilized to a constant weight. These dry powders were as PBOM. The extraction yield of PBOM was calculated as the following formula:

(1) $$\begin{array}{c} \text{extraction yield}\left(\%\right)=\frac{W_{1}}{w_{0}}\times 100, \end{array}$$

where *W*_1_ is the weight of PBOM (g) and *w*_0_ is the weight of dried pretreatment powder from *Buddleja officinalis* (g).

### 2.3. Design of PBOM Extraction Parameter Optimization by Response Surface Method

Through the single factor optimization experiment, it was found that the main factors affecting the extraction yield of PBOM were as follows: ratio of water to raw material, ultrasonic power, ultrasonic time, and ultrasonic temperature. From this foundation, we further employed the Box–Behnken Design (BBD), a commonly used method for response surface experimental technology, to optimize the optimal extraction parameters of PBOM. In the BBD implementation process of PBOM extraction parameter optimization, the extraction yield (%) of PBOM was used as the response index, while the ratio of water to raw material (*X*_1_), ultrasonic power (*X*_2_), ultrasonic time (*X*_3_), and ultrasonic temperature (*X*_4_) were seen as the variables. Based on the design principle of BBD, the three levels selected for each variable were encoded according to formula (2). The encoded value of the independent variable and the specific level were displayed in Table 1. The whole experimental scheme was composed of 29 independent experimental groups. All data from each independent experimental group were used to fit the second-order polynomial model of the following formula:

(2) $$\begin{array}{c} x_{i}=\frac{\left(X_{i}-X_{0}\right)}{\Delta X_{i}}, \end{array}$$

(3) $$\begin{array}{c} Y=\alpha_{0}+\sum_{i=1}^{3}\alpha_{i}X_{i}+\sum_{i=1}^{3}\alpha_{ii}X_{i}^{2}+\sum_{i=1}^{2}\sum_{j=i+1}^{3}\alpha_{ij}X_{i}X_{j}, \end{array}$$

where *x*_*i*_ was the coded value of each independent variable, *X*_*i*_ was the actual value of each independent variable, *X*_0_ was the actual value of each independent variable at center point, and Δ*X*_*i*_ was the step change value of each independent variable. *Y* was the predicted response (yield of PBOM), *α*_0_, *α*_*i*_, *α*_*ii*_, and *α*_*ij*_ were the regression coefficients for intercept, linear, quadratic, and interaction terms, respectively, and *X*_i_ and *X*_*j*_ were the independent variables (*i* ≠ *j*).

### 2.4. Purification Scheme Design of APBOM

To obtain APBOM, the DEAE-52 cellulose and Sephadex G-100 gel column chromatography were adopted further to isolate and purify the PBOM. Briefly, 1 ml PBOM solution with a concentration of 100 mg/ml was uploaded into the DEAE-52 cellulose column (2.6 × 50 cm). In order to screen the optimal NaCl concentration for gradient elution, a linear gradient elution was first implemented. In the linear gradient elution process, the column was sequentially eluted as the following procedure at a flow rate of 1 ml/min: deionized water ran from 0 to 200 min; 0 ⟶ 2.0 M NaCl solution operated from 200–1000 min. The eluent was automatically collected as 10 ml per tube. The carbohydrate content in each tube was determined using the phenol-sulfuric acid method. The elution curve was prepared when the absorbance of each tube, concentration of NaCl, and ordinal number of the eluent collected were used as the main ordinate, secondary ordinate, and abscissa, respectively. The NaCl concentration corresponding to the end point of each elution peak was taken as that of gradient elution. Then, the gradient elution of the column was carried out at the optimum eluent concentration. The elution curve was drawn using the same method mentioned above. The eluents under the same eluent peak were combined, concentrated, dialyzed, and lyophilized to gain the purified component. The electrical characteristics of each purified component were evaluated by a zeta potential determinator. 1 ml purified components (100 mg/ml) with strong electronegativity and high yield were uploaded into the Sephadex G-100 column (1.6 × 60 cm) to further purify. After loading, the Sephadex G-100 column was eluted with 0.25 M sodium nitrate solution at a flow rate of 1 ml/min. The eluent was synchronously collected with 10 ml per tube by an automatic collector. The carbohydrate content of the eluent was determined using the same method as above. Taking the absorbance of each tube as the ordinate and the serial number of eluents as the abscissa, the eluent curve was drawn. Each tube under the same elution peak with the maximum peak area was collected, concentrated, dialyzed, and lyophilized. This freeze-dried sample would be offered as APBOM.

### 2.5. Characterization Method of APBOM

#### 2.5.1. Chemical Analyses

Total polysaccharide content of APBOM was determined with D-glucose as the standard by the phenol-sulfuric acid method [19]. The Coomassie brilliant blue staining was used to measure the protein content of APBOM using the bovine serum albumin as reference [20]. The content of the sulphated group in polysaccharides was tested by the barium chloride-gelatin method using K_2_SO_4_ as the standard [21]. The sulfuric acid-m-hydroxyl biphenyl method was hired to evaluate the uronic acid content of this polysaccharide [10].

#### 2.5.2. Analysis of UV-Visible Spectra

10.0 mg APBOM was dissolved in 1.0 mL ultrapure water. The UV-visible spectra of APBOM solution in the range of 200–800 nm were collected by a UV-2450 Spectrophotometer (Shimadzu Co., Kyoto, Japan) at 25°C using ultrapure water as the reference.

#### 2.5.3. Measurement of FT-IR Spectra

KBr powder, 100 mg, was added into 1 mg of absolutely dried APBOM. After mixing thoroughly in an agate mortar, the mixed powder was pressed into a circular tablet. A Nicolet 6700 FT-IR Spectrometer (Thermo Co., USA) was adopted to scan its infrared spectra in the frequency range of 4000–400 cm^−1^.

#### 2.5.4. Determination of Monosaccharide Composition

The monosaccharide composition of APBOM was tested by high-performance liquid chromatography (HPLC) after precolumn derivatization. Briefly, 5 mg of APBOM was hydrolyzed with 10 ml of 2.0 M trifluoroacetic acid (TFA) at 120°C in an oven for 4 h. The remaining TFA was completely removed by repeated evaporation with methanol. The residue was dissolved in 1 mL distilled water to obtain a hydrolyzed sample solution. Then, the hydrolyzed sample solution was derivatized by 1-phenyl-3-methyl-5-pyrazolone (PMP) according to the reported method [22]. The derivatives were analyzed by HPLC by using the C18 column (4.6 × 250 mm, 5 *μ*m, Shimadzu, Japan) as the separator column and ultraviolet (UV) detector. At a flow rate of 1.0 ml/min and column temperature of 35°C, the column was eluted by mobile phase, which composed of 82.0% phosphate buffer solution (0.1 M, pH 7.0) and 18.0% acetonitrile (v/v). The detection wavelength was set to 245 nm. Similarly, the monosaccharide standards were derivatized by PMP and analyzed by HPLC using the same chromatographic conditions as above. The monosaccharide composition of APBOM was identified by comparing with the retention time of standard monosaccharide peak. In addition, the content of each monosaccharide was quantified by bringing their peak area into the standard curve of the monosaccharide standards.

#### 2.5.5. Evaluation of Homogeneity and *M*_*w*_

The homogeneity and *M*_*w*_ of APBOM were evaluated by using a size-exclusion HPLC chromatography (HPGPC) instrument (Agilent 1200, USA) with a refractive index detector (RID). The chromatographic analysis was performed by a TSK-GEL G3000SW_xl_ gel-filtration chromatographic column (7.5 × 300 mm, 5 *μ*m, Tosoh Corp., Japan). The column was eluted with 0.1 M Na_2_SO_4_ solution in PBS buffer (0.01 M, pH 6.8) with a flow rate of 0.8 ml/min at 25°C. The standard curve was prepared by a series of standard dextrans (5.2, 23.8, 48.6, 148, 273, 410, and 668 kDa). The calibration curves were drawn by plotting the retention time against the logarithm of their respective *M*_*w*_. A chromatographic peak with symmetry and singleness profile was considered as an indicator of the APBOM homogeneity. The *M*_*w*_ was calculated by substituting the retention time into the equation of the calibration curve.

### 2.6. Measurement of APBOM Antiangiogenic Activities

#### 2.6.1. Cell Culture

Human umbilical vein endothelial cells (HUVECs) were bought from Lonza (San Diego, CA). HUVECs were primarily cultured in an EGM™-2 BulletKit™ medium of Lonza (San Diego, CA) according to the manufacturer's protocol. HUVECs reached exponential phase were chosen for further assay.

#### 2.6.2. Cell Proliferation Assay of HUVECs

The MTT colorimetric assay was used to evaluate the effect of APBOM on HUVEC proliferation. In brief, HUVECs of the exponential phase were adjusted to a density of 1 × 10^5^ cells/ml and then inoculated in a 96-well plate with 100 *μ*l per well. The cells were cultured in a humidified atmosphere of 5% CO_2_ at 37°C for 2 h. Subsequently, the medium of the cells was removed and transformed into culture media which contained various concentrations (0, 20, 40, 80, 160, 320, or 640 *μ*g/ml) of APBOM to incubate for another 12 h. The control wells and blank wells were also cultured by using the same methods. After the completion of the incubation, the cells were washed with PBS and then treated with MTT kits according to the manufacturer's guidelines. The cell proliferation activity of APBOM on HUVECs was evaluated by its cell viability (%), which was calculated with the reported method [10].

#### 2.6.3. Transwell Migration Experiment of HUVECs

Transwell migration assay of HUVECs was carried out by transwell chambers (Corning Life Sciences, Tewksbury, MA, USA). Briefly, 100 *μ*l of HUVECs with a density of 1 × 10^5^ in 1% FBS was seeded into the upper chambers. Meanwhile, a 600 *μ*l culture medium containing 10% FBS was added into the bottom chambers. After the cells attached to the wall, APBOM solutions were loaded into the upper chambers. Under the same reaction conditions, the equal volume of blank medium was used as the control group. After incubation for 96 h, the nonmigrated cells that stayed on the top surface of the polycarbonate membrane were completely cleaned off. 800 *μ*l of 4.0% paraformaldehyde was used to fix the migrated cells attaching to the opposite side of the polycarbonate membrane for 20 min. The fixed cells were stained for 2-3 min by using 800 *μ*L of 0.1% crystal violet cell colony kit and then photographed at random by an inverted microscope (CK40-F200, Olympus, Tokyo, Japan). The cells on the membrane were counted in five random microscopic fields. The migration rate (%) was considered as transwell migration ability. The migration rate (%) was calculated as the following formula:

(4) $$\begin{array}{c} \text{migration rate }\left(\%\right)=\frac{N_{S}}{N_{c}}\times 100\%, \end{array}$$

where *N*_*S*_ and *N*_*c*_ were the number of the cells in APBOM groups and control groups, respectively.

#### 2.6.4. Matrigel-Based Tube Formation Assay of HUVECs

The inhibitory effect of APBOM on HUVECs tube formation was tested by Matrigel-based tube formation experiments. The assays were performed as the reported method [10, 23].

### 2.7. Statistical Analysis

All experiments were implemented at least three times independently. The one-way analysis of variance and Student's *t*-test were used to analyze the resulting data. All measurement data were expressed as the mean ± standard deviation $\left(\bar{x}\pm s\right)$. Values of *P* < 0.05 were accounted as be statistically significant.

## 3. Results

### 3.1. Optimization of PBOM Extraction Parameter

#### 3.1.1. Model Fitting of PBOM Extraction

The response surface experiment scheme and results for PBOM extraction were summarized in Table 1. All outcome data in Table 1 were fitted with multiple regression analysis by the software of Design Expert version 7.0. As a result, the fitted model used for the extraction yield (%) of PBOM to predict the relationship between the independent variables and the dependent variable could be expressed as the following formula:

(5) $$\begin{array}{c} Y=-25.79485+0.50583X_{1}+0.017663X_{2}+0.21647X_{3}+0.57148X_{4}-0.000005X_{1}X_{2}-0.00000333X_{1}\;X_{3}+0.00005X_{1}X_{4}+0.000055X_{2}X_{3}-0.0000075X_{2}X_{4}-0.0001X_{3}X_{4}-0.009846X_{1}^{2}-0.00004061X_{2}^{2}-0.002455X_{3}^{2}-0.0045741X_{4}^{2}, \end{array}$$

where *Y* represented the extraction yield (%) of PBOM, and *X*_1_, *X*_2_, *X*_3_, and *X*_4_ meant the ratio of water to raw material, ultrasonic power, ultrasonic time, and ultrasonic temperature, respectively.

#### 3.1.2. ANOVA for Regression Equation of Response Surface Model and Interaction Analysis of Each Factor

ANOVA is often hired to assess the significance and suitability of the model. A statistical summary of the ANOVA for PBOM extraction is shown in Table 2. As depicted in Table 2, the model *F* value of 42.528 together with a very low probability *P* value (*<*0.0001) implied that this model was the high significance. For the model fitting, the coefficient of determination (*R*^2^) was 0.9769, suggesting that only 2.31% of the total variation was not explained by this model. The insignificant *F* value from the lack of fit (*P*=0.0600 > 0.05) confirmed the validity of this model. The adjusted determination coefficient of 0.9541 also demonstrated that the model was highly significant. According to the size of the *F* value of each factor in Table 2, it can be seen clearly that the order of influence from each factor on the extraction yield (%) of PBOM was ultrasonic time, ultrasonic power, ultrasonic temperature, and ratio of water to raw material. Among them, the effect of ultrasonic time, ultrasonic power, and ultrasonic temperature on the extraction yield (%) of PBOM reached an extremely significant level (*P* < 0.0001).

**Figure 1:**
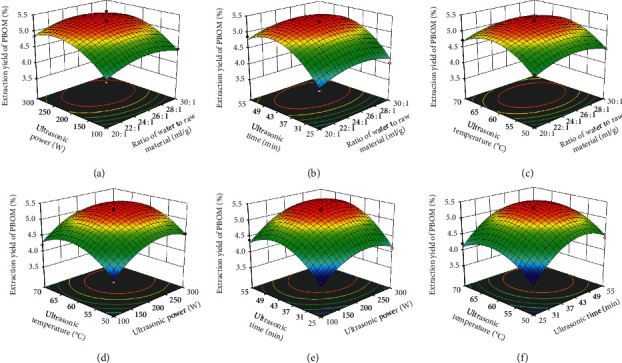
Response surface plots showing the interactive effects from the ratio of water to raw material, ultrasonic power, ultrasonic time, and ultrasonic temperature on extraction yield of PBOM.

**Figure 2:**
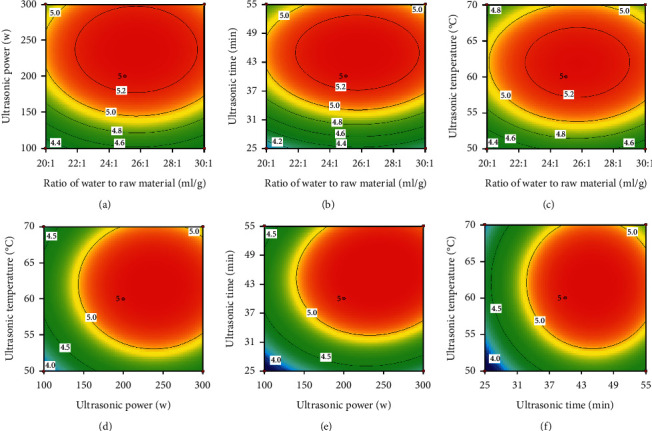
Contour plots showing the interactive effects from the ratio of water to raw material, ultrasonic power, ultrasonic time, and ultrasonic temperature on extraction yield of PBOM.

As shown in Figures 1 and 2 and Table 2, the interactions between the factors appeared to be insignificant, indicating that the influence of various factors on extraction yield (%) of PBOM would represent a linear relationship.

#### 3.1.3. Verification of the Predictive Model and Determination of Optimal Parameter Conditions

The optimal theoretical parameter conditions of PBOM extraction were predicted by Using Design Expert version 7.0 as follows: ratio of water to raw material of 25.71 : 1, ultrasonic power of 240.84 W, ultrasonic time of 45.35 min, and ultrasonic temperature of 61.91°C. Under these extraction conditions, the extraction yield (%) of PBOM could reach the maximum value of 5.43% in theory. Taking into account the feasibility of practical operation, the parameter conditions were adjusted appropriately as follows: ratio of water to raw material of 26 : 1, ultrasonic power of 240 W, ultrasonic time of 45 min, and ultrasonic temperature of 62°C. Under these conditions, the actual yield of PBOM was 5.39 ± 0.16%. The standard deviation compared to the model's predicted extraction yield was 0.369%. The results implied that the model equation can well matched with the predicted values and be used for practical prediction.

### 3.2. Purification of APBOM

**Figure 3:**
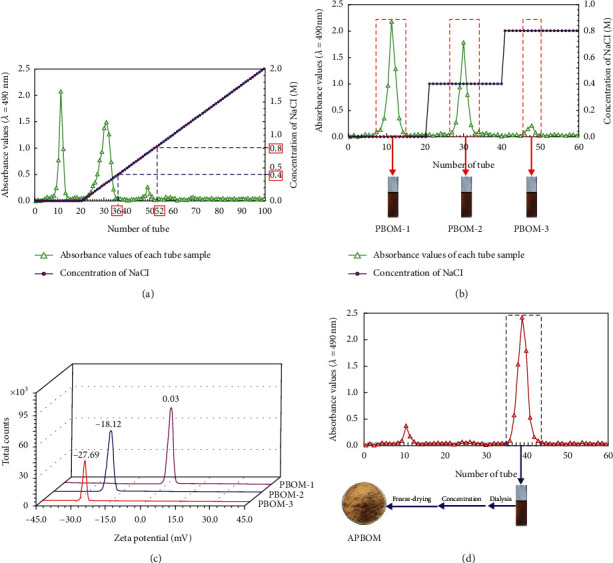
The elution curve of PBOM from DEAE-52 column chromatography by linear gradient elution with 0–2 M NaCl (a), the elution curve of PBOM from DEAE-52 column chromatography (b), zeta potential analysis of PBOM-1, PBOM-2, and PBOM-3 (c), and the elution curve of PBOM-2 by Sephadex G-100 column chromatography (d).

As shown in Figure 3(a), the purified elution curve of PBOM revealed three distinct elution peaks by DEAE-52 cellulose column chromatography together with linear elution of NaCl. The results indicated that three purified components could be obtained from the purification of PBOM by DEAE-52 cellulose column chromatography separation. According to the distribution of elution peaks from Figure 3(a), the elution of NaCl resulted in two elution peaks and the corresponding NaCl concentrations at the end points of the two elution peaks were 0.4 M and 0.8 M, respectively. The data suggested that the compositions of the gradient elution solution for the purification of PBOM by DEAE-52 cellulose column chromatography were NaCl solution of 0 M (deionized water), 0.4 M, and 0.8 M, respectively. Using these gradient eluents, a gradient eluent experiment of PBOM purification by DEAE-52 cellulose column chromatography was carried out, and its elution curve is provided in Figure 3(b). As shown in Figure 3(b), three narrow and distinct elution peaks appeared on the elution curve. All eluents in each tube under each eluent peak were combined and then concentrated, dialyzed, and freeze-dried to get the three purified components. For the convenience of analysis, the three purified components are named as PBOM-1, PBOM-2, and PBOM-3 according to the order of peak time from small to large. On this basis, the zeta potential of each purified component was determined and is presented in Figure 3(c). Figure 3(c) discloses that both PBOM-2 and PBOM-3 were electronegative, indicating that they were acidic polysaccharides. However, we knew from Figure 3(b) that the yield of PBOM-3 was too low to be collected and further studied. Because of this, PBOM-3 was abandoned, instead of PBOM-2 to carry out further research. As shown in Figure 3(d), PBOM-2 was further purified by a Sephadex G-100 column to appear two polysaccharide peaks on the elution curve. Nonetheless, the peak area of the second elution peak was much larger than that of the first. Hence, all eluents in each tube from the second elution peak was collected, concentrated, dialyzed, and lyophilized to provide target polysaccharide APBOM. By calculating the yield of APBOM, it was found that its yield (%) was as high as 9.57 ± 0.76% relative to PBOM.

### 3.3. Characterization of APBOM

Chemical analysis revealed that total polysaccharide content of APBOM was 94.37 ± 1.14%. There was not any protein in APBOM. However, its uronic acid content was as high as 17.41 ± 0.35%. In addition, APBOM also contained 1.68 ± 0.17% sulfuric acid group content.

**Figure 4:**
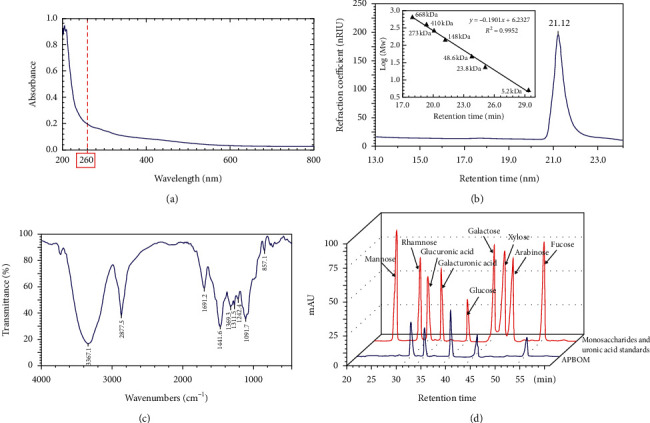
The characterization of APBOM: UV-vis spectrum (a), HPGPC and standard curve of molecular weight determination (b), FT-IR spectrum (c), and HPLC chromatogram for monosaccharide composition analysis with PMP derivatization (d).

As shown in Figure 4(a), the UV-vis spectrum of APBOM had no absorption peak at 260–280 nm, confirming again the absence of protein in APBOM. The results were consistent with those of chemical analysis. Furthermore, an independent symmetrical absorption peak of APBOM from HPGPC (Figure 4(b)) demonstrated that it was a homogeneous polysaccharide on molecular weight distribution and its mean *M*_*w*_ was calculated to be 165.4 kDa. And on this basis, FT-IR spectra of Figure 4(c) found the characteristic absorption peaks of APBOM were distributed at 3367.1 cm^−1^, 2877.5 cm^−1^, 1691.2 cm^−1^, 1441.6 cm^−1^, 1369.3 cm^−1^, 1311.5 cm^−1^, 1242.4 cm^−1^, 1091.7 cm^−1^, and 857.1 cm^−1^. Among them, the strong absorption peak at 3367.1 cm^−1^ was attributed to the stretching vibration of the O-H bond. The characteristic absorption peak from the stretching vibration and deformation vibration absorption peaks of the C-H bond was identified based on the absorption peaks at 2877.5 cm^−1^, 1369.3 cm^−1^, and 1311.5 cm^−1^. And more importantly, the asymmetric and symmetric vibrations of the C=O bond also found the absorption peaks at 1691.2 cm^−1^ and 1441.6 cm^−1^. Meanwhile, the absorption peaks at 1242.4 cm^−1^ were assigned to asymmetric vibrations of S-O. Furthermore, the absorption peak at 852.7 cm^−1^ suggested that there was the *α*-glucosidine bond or S-O-S symmetric stretching vibration. Taking together, the characteristic absorption peaks of APBOM indicated that it was an acidic polysaccharide. In addition, the HPLC results of the monosaccharide component analysis (Figure 4(d)) revealed that the APBOM was a typical heteropolysaccharide and was composed of glucose, galactose, fucose, glucuronic acid, and galacturonic acid in the molar ratio of 6.75 : 3.33 : 1.79 : 1.42 : 1.00. The appearance of the glucuronic acid and galacturonic acid peaks in Figure 4(d) confirmed once again that APBOM was an acidic polysaccharide.

### 3.4. Antiangiogenic Activities of APBOM

#### 3.4.1. Effect of APBOM on HUVEC Proliferation

**Figure 5:**
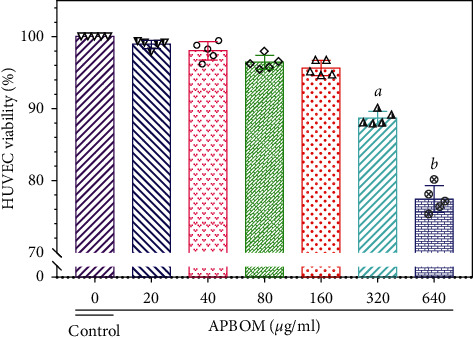
Effects of APBOM on viability of HUVECs. Data represent mean ± SD for three independent experiments. Superscript letters *a* and *b*, respectively, designate significant differences, *P* <0.05 and *P* <0.01, compared with the model group.

As shown in Figure 5 an inhibitory activity of the APBOM for HUVEC proliferation was clearly observed. And this inhibition exhibited a significant dose-dependent manner. It also was found that the effect of APBOM concentration of 0–160 *μ*g/ml on HUVEC proliferation was not significant. However, APBOM showed a significant inhibitory effect on HUVEC proliferation when its concentration increased to 320 *μ*g/ml. These results suggested that APBOM processed certain inhibitory activities on HUVEC proliferation without resulting in significant cytotoxicity, as long as the concentration is no more than 160 *μ*g/ml. Therefore, the concentrations of 0–160 *μ*g/ml APBOM were picked to perform further experiment.

#### 3.4.2. Effect of APBOM on HUVEC Transwell Migration

**Figure 6:**
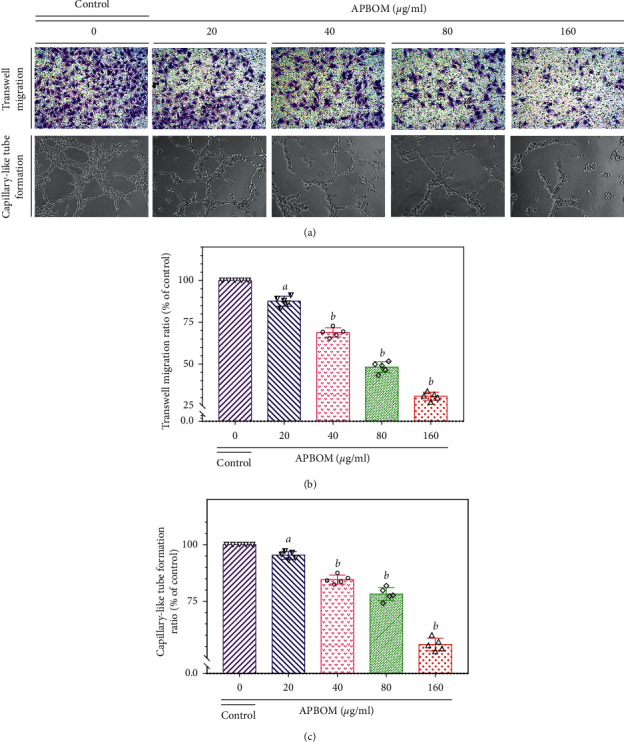
Representative photographs of APBOM on the transwell migration and the capillary-like tube formation of HUVECs (a), quantitative analysis of the transwell migration rate (%) (b), and capillary-like tube formation rate (%) (c) by different concentrations of APBOM-treated HUVECs. Data represent mean ± SD for three independent experiments. Superscript letters *a* and *b*, respectively, designate significant differences, *P* <0.05 and *P* <0.01, compared with the model group.

Effect of APBOM on HUVEC transwell migration is depicted in Figure 6. As shown in Figure 6(a), a large number of HUVECs migrated to the bottom chambers in the control group. However, this migration of HUVECs was significantly inhibited after APBOM intervention. As the concentration of APBOM increased, the number of migrated HUVECs showed a dose-dependent decrease. After intervention by APBOM of 20, 40, 80, and 160 *μ*g/ml, the migration rate (%) of HUVECs was respectively reduced to 87.56%, 68.81%, 47.99%, and 27.17% relative to the control group (Figure 6(b)). When the concentration of APBOM reached 160 *μ*g/ml, only a few HUVECs migrated to the bottom chambers. Since the maximum concentration of APBOM intervention was only 160 *μ*g/ml, it could be ruled out that the decreased migration rate of HUVECs was caused by the cytotoxicity of the drug. These results fully demonstrated that APBOM could significantly inhibit the migration of HUVECs.

#### 3.4.3. Effect of APBOM on Matrigel-Based Tube Formation of HUVECs

As shown in Figure 6(a), a representative tube image in the control group was obviously observed. Unlike that, the numbers of unabridged tubes in APBOM intervention groups were gradually reduced and significantly less than those of the control group. With the increase of APBOM concentration, it became more and more difficult to find the unabridged tubes. The capillary-like tube formation ratio (%) was gradually to reduce in a dose-dependent manner (Figure 6(c)). After APBOM concentration reached 80 *μ*g/ml, there were only incomplete tubular structures (Figure 6(a)). The capillary-like tube formation ratio (%) from treatment of 80 and 160 *μ*g/ml APBOM was, respectively, slipped below 78.14% and 55.95% of the control group (Figure 6(c)). Similarly, the concentration of APBOM used in this experiment was safe without cytotoxicity. The inhibitory effect of APBOM on Matrigel-based tube formation of HUVECs also eliminated its own cytotoxicity. Thus, the data confirmed that APBOM could markedly suppress the tube formation of HUVECs.

## 4. Discussion

Angiogenesis refers to the process of generating new capillaries from preexisting vessels in bud or nonbud form by proliferation and migration of vascular endothelial cells based on existing capillaries [24]. Clinical data have shown that the neovascularization derived from angiogenesis was brittle and prone to bleeding, leakage, edema, and other pathological features, resulting in scar formation and blindness, which plays an extremely critical role in many blinding eye diseases [2]. Currently, inhibition of angiogenesis was considered as an important approach for the treatment of many eye diseases [4, 5]. A growing number of studies have suggested that acidic polysaccharides have significant antiangiogenic activity [9, 12]. Meanwhile, *Buddleja officinalis* has been widely used in the treatment of many eye diseases caused by angiogenesis. Based on these facts, we naturally came to such a speculation that the effective substances of *Buddleja officinalis* for the treatment of neovascularization eye disease were the presence of its acidic polysaccharides [15, 25, 26]. Encouragingly, the research in this paper confirmed our theoretical hypothesis. In the present paper, APBOM was not only successfully obtained from *Buddleja officinalis*, but also proved to have significant antiangiogenic activity.

Because of the existence of acidic groups, their physical and chemical properties have changed greatly from that of nonacidic groups [8]. Therefore, there are many special requirements for the extraction of these polysaccharides. The extraction of acidic polysaccharides should be rapid and completely carried out on the premise of not destroying its acidic groups and glycosidic bonds. At present, the extraction methods of acid polysaccharides mainly include hot water extraction, alkaline water extraction, enzyme-assisted extraction, ultrasound-assisted extraction, and microwave-assisted extraction [27]. Among them, ultrasound-assisted extraction has been widely praised by many scholars for its function of both destroying cell wall to promote mass transfer and accelerating dissolution. More and more studies indicated that ultrasonic-assisted technology can not only save time and cost but also get higher yield [28]. It is exactly based on this consideration that ultrasonic-assisted extraction in the present paper was used to extract PBOM. The results suggested that the extraction yield (%) of PBOM was as high as 5.39 ± 0.16% by ultrasonic-assisted extraction. Its extraction yield (%) was 38.56% higher than that of hot water extraction of Mu et al. [29], which implied that ultrasonic-assisted extraction was an excellent method for PBOM extraction.

The sulfuric acid-carbazole method and sulfuric acid-m-hydroxybiphenyl way are commonly used for the determination of uronic acid. However, the determination of uronic acid in polysaccharides by the sulfuric acid-carbazole method often has a large positive deviation because of glucose and some other neutral polysaccharides reacted with carbazole easily [27]. This error can be avoided by sulfuric acid-m-hydroxybiphenyl method, so it is a common method for the determination of uronic acid in polysaccharides. Even so, the sulfuric acid-m-hydroxybiphenyl method can only determine the content of uronic acid and cannot identify its type [27]. In recent years, many scholars began to use HPLC and GC to simultaneously determine and identify uronic acid in polysaccharides. In this study, the content of uronic acid in APBOM was determined to be 17.41 ± 0.35% by the sulfuric acid-m-hydroxybiphenyl method, and their type was further identified by HPLC to be composed of glucuronic acid and galacturonic acid with the molar ratio of 1.42 : 1.00.

The proliferation, chemotaxis, migration, and lumen formation of vascular endothelial cells are the initial and central links of angiogenesis [10]. Based on this spot, the evaluation of angiogenesis *in vitro* is mainly simulated by experiments on proliferation, migration, and lumen formation of HUVECs at present [30]. In this paper, we systematically investigated the effects of APBOM on endothelial cell proliferation, migration, and lumen formation of HUVECs by MTT, transwell chambers, and Matrigel-based tube formation assay, respectively. Theoretically, if we want to prove that APBOM could inhibit angiogenesis, it must be observed that APBOM could significantly suppress the proliferation, migration, and lumen formation of HUVECs. Our results showed that APBOM can indeed markedly inhibit the proliferation, migration, and lumen formation of HUVECs, indicating that APBOM has an antiangiogenic effect.

In conclusion, the extraction, purification, characterization, and antiangiogenic activity of APBOM were systematically studied at the present paper. The results showed that the optimal parameter conditions for PBOM extraction were as follows: ratio of water to raw material of 26 : 1, ultrasonic power of 240 W, ultrasonic time of 45 min, and ultrasonic temperature of 62°C. Under this optimal condition, the yield of PBOM was as high as 5.39 ± 0.16%. On this basis, APBOM was obtained with the yield of 9.57 ± 0.76% (relative to PBOM) by applying DEAE-52 cellulose and Sephadex G-100 gel column chromatography together. Further characterization revealed that APBOM was an acidic polysaccharide with the total polysaccharide content of 94.37 ± 1.14%, sulfuric acid group of 1.68 ± 0.17%, and uronic acid content of 17.41 ± 0.35%. Its mean *M*_*w*_ was 165.4 kDa and was composed of glucose, galactose, fucose, glucuronic acid, and galacturonic acid in the molar ratio of 6.75 : 3.33 : 1.79 : 1.42 : 1.00. In addition, APBOM had a significant antiangiogenesis *in vitro*.

## Figures and Tables

**Table 1:** Experimental schemes and results of Box–Behnken design for PBOM extraction.

Serial number	Ratio of water to raw material (ml/g)	Ultrasonic power (*W*)	Ultrasonic time (min)	Ultrasonic temperature (°C)	Extraction yield of PBOM (%)
	*X*_1_ (code *x*_1_)	*X*_2_ (code *x*_2_)	*X*_3_ (code *x*_3_)	*X*_4_ (code *x*_4_)	
1	20 : 1 (−1)	100 (−1)	40 (0)	60 (0)	4.17
2	30 : 1 (1)	100 (−1)	40 (0)	60 (0)	4.45
3	20 : 1 (−1)	300 (1)	40 (0)	60 (0)	4.85
4	30 : 1 (1)	300 (1)	40 (0)	60 (0)	5.12
5	25 : 1 (0)	200 (0)	25 (−1)	50 (−1)	3.79
6	25 : 1 (0)	200 (0)	55 (1)	50 (−1)	4.40
7	25 : 1 (0)	200 (0)	25 (−1)	70 (1)	4.21
8	25 : 1 (0)	200 (0)	55 (1)	70 (1)	4.76
9	20 : 1 (−1)	200 (0)	40 (0)	50 (−1)	4.39
10	30 : 1 (1)	200 (0)	40 (0)	50 (−1)	4.46
11	20 : 1 (−1)	200 (0)	40 (0)	70 (1)	4.73
12	30 : 1 (1)	200 (0)	40 (0)	60 (0)	4.81
13	25 : 1 (0)	100 (−1)	25 (−1)	60 (0)	3.86
14	25 : 1 (0)	300 (1)	25 (−1)	60 (0)	4.13
15	25 : 1 (0)	100 (−1)	55 (1)	60 (0)	4.39
16	25 : 1 (0)	300 (1)	55 (1)	60 (0)	4.99
17	20 : 1 (−1)	200 (0)	25 (−1)	60 (0)	3.96
18	30 : 1 (1)	200 (0)	25 (−1)	60 (0)	4.04
19	20 : 1 (−1)	200 (0)	55 (1)	60 (0)	4.87
20	30 : 1 (1)	200 (0)	55 (1)	60 (0)	4.94
21	25 : 1 (0)	100 (−1)	40 (0)	50 (−1)	3.84
22	25 : 1 (0)	300 (1)	40 (0)	50 (−1)	4.57
23	25 : 1 (0)	100 (−1)	40 (0)	70 (1)	4.22
24	25 : 1 (0)	300 (1)	40 (0)	70 (1)	4.92
25	25 : 1 (0)	200 (0)	40 (0)	60 (0)	5.28
26	25 : 1 (0)	200 (0)	40 (0)	60 (0)	5.20
27	25 : 1 (0)	200 (0)	40 (0)	60 (0)	5.33
28	25 : 1 (0)	200 (0)	40 (0)	60 (0)	5.32
29	25 : 1 (0)	200 (0)	40 (0)	60 (0)	5.29

**Table 2:** ANOVA and the significance test of the response surface quadratic model for PBOM extraction.

Variables	Sum of squares	Df	Mean square	*F* value	*P* value
Model	6.438	14	0.459	42.528	<0.0001^*b*^
*X*_1_	0.061	1	0.061	5.567	0.0333^*a*^
*X*_2_	1.111	1	1.111	102.665	<0.0001^*b*^
*X*_3_	1.584	1	1.584	146.491	<0.0001^*b*^
*X*_4_	0.403	1	0.403	37.298	<0.0001^*b*^
*X*_1_, *X*_2_	2.5*E* − 05	1	2.5*E* − 05	0.003	0.9623^*c*^
*X*_1_, *X*_3_	2.5*E* − 05	1	2.5*E* − 05	0.003	0.9623^*c*^
*X*_1_, *X*_4_	2.5*E* − 05	1	2.5*E* − 05	0.003	0.9623^*c*^
*X*_2_, *X*_3_	0.027	1	0.027	2.518	0.1349^*c*^
*X*_2_, *X*_4_	0.001	1	0.001	0.021	0.8874^*c*^
*X*_3_, *X*_4_	0.001	1	0.001	0.083	0.7772^*c*^
*X*_1_^2^	0.393	1	0.393	36.348	<0.0001^*b*^
*X*_2_^2^	1.071	1	1.071	98.954	<0.0001^*b*^
*X*_3_^2^	1.979	1	1.979	183.046	<0.0001^*b*^
*X*_4_^2^	1.357	1	1.357	125.502	<0.0001^*b*^
Residual	0.151	14	0.011		
Lack of fit	0.141	10	0.014	5.356	0.0600^*c*^
Pure error	0.011	4	0.003		
Corrected total	6.589	28			

*R* ^2^ = 0.9769; adjusted *R*^2^ = 0.9541. ^*a*^5% significance level. ^*b*^1% significance level. ^*c*^Not significant relative to the pure error.

## Data Availability

All the data used to support the findings of this study are available from the corresponding author upon reasonable request.
